# Peripheral and central nervous system toxicities of Bruton tyrosine kinase Inhibitors: a real-world pharmacovigilance study

**DOI:** 10.3389/fmed.2026.1903660

**Published:** 2026-09-15

**Authors:** Jianxun Zhang

**Affiliations:** The National Police University for Criminal Justice, Baoding, China

**Keywords:** acalabrutinib, Bruton’s tyrosine kinase inhibitor, FAERS, ibrutinib, nervous system disorders adverse events, pharmacovigilance, zanubrutinib

## Abstract

**Background:**

Bruton’s tyrosine kinase (BTK) inhibitors have revolutionized the management of B-cell malignancies, yet emerging evidence indicates that they may be associated with nervous system disorders adverse events (AEs). Given their expanding therapeutic applications, especially in nervous system disorders, understanding their neurotoxicity profiles is of critical clinical importance.

**Methods:**

We conducted a retrospective pharmacovigilance analysis using data from the United States. FDA Adverse Event Reporting System (FAERS) covering Q1 2013 to Q4 2024. Reports listing acalabrutinib, ibrutinib, or zanubrutinib as the primary suspect drug were extracted. Nervous system disorder AEs were identified based on MedDRA System Organ Class classification. Disproportionality analyses were performed using reporting odds ratio (ROR) and proportional reporting ratio (PRR), and multivariate logistic regression was applied to adjust for confounders, including age, sex, and weight. Time-to-onset distributions, severity patterns, and mortality rates were also evaluated.

**Results:**

Out of 75,240 BTK inhibitor-associated AE reports, 10,435 (13.87%) were coded to the MedDRA System Organ Class (SOC) “Nervous system disorders.” Ibrutinib contributed the largest absolute number of neurological AE reports (*n* = 9,097), followed by acalabrutinib (*n* = 1,129) and zanubrutinib (*n* = 209). The most frequently reported events included headache, dizziness, cerebrovascular accident, loss of consciousness, and peripheral neuropathy. At the SOC level, however, no overall positive disproportionality signal was observed for any of the three agents (acalabrutinib ROR = 0.95, 95% CI 0.90–1.01; ibrutinib ROR = 0.68, 95% CI 0.67–0.69; zanubrutinib ROR = 0.61, 95% CI 0.54–0.69). Positive signals were instead identified for selected preferred terms, including clinically important haemorrhagic and cerebrovascular events. Reported fatal outcomes were most frequent among reports containing cerebrovascular accident and cerebral hemorrhage; these findings should be interpreted cautiously because FAERS does not adjudicate cause of death.

**Conclusion:**

In this FAERS-based pharmacovigilance study, BTK inhibitors showed distinct reporting patterns of nervous system disorders AEs, particularly at the preferred-term and HLGT levels, rather than an overall SOC-level disproportionality signal. Ibrutinib contributed the largest number of neurological AE reports, whereas comparisons involving zanubrutinib require caution because of sparse reporting and shorter market exposure. These findings support careful neurological monitoring and individualized risk assessment, but they should not be interpreted as evidence of causality.

## Introduction

The development of small-molecule inhibitors targeting Bruton’s tyrosine kinase (BTK) represents a significant therapeutic advancement in the management of various B-cell hematologic malignancies, including chronic lymphocytic leukemia, mantle cell lymphoma, and Waldenström’s macroglobulinemia (1–3). As a key component of the Tec kinase family, BTK functions as a non-receptor tyrosine kinase predominantly found in B-lymphocytes and diverse myeloid cell populations, playing a crucial role in modulating cellular growth, apoptosis resistance, and maturation processes via B-cell receptor-mediated signaling cascades. The therapeutic landscape now includes multiple clinically validated BTK inhibitors, with ibrutinib serving as the pioneering first-generation compound, followed by next-generation alternatives such as acalabrutinib and zanubrutinib.

Of concern, BTK inhibitors can induce a range of nervous system disorders adverse events (AEs) during treatment (4). Clinical studies have reported that patients receiving BTK inhibitor therapy may experience neurological symptoms such as headache, dizziness, fatigue, and peripheral neuropathy (5, 6). Notably, recent reports have described a rare but severe adverse reaction of aseptic meningitis associated with zanubrutinib (7).

The FDA Adverse Event Reporting System (FAERS), as one of the largest post-marketing drug safety surveillance databases worldwide, provides important data resources for monitoring drug safety (8, 9). Pharmacovigilance studies based on the FAERS database can identify rare adverse reactions not detected in clinical trials and provide real-world evidence for benefit–risk evaluation (10, 11). Given the potential value of BTK inhibitors in the treatment of nervous system disorders and the possibility of nervous system disorders AEs, a study on the neurological safety of BTK inhibitors based on the FAERS database is of significant clinical importance.

This study aims to systematically analyze the neurological safety profiles of different BTK inhibitors, identify potential safety signals, and guide rational clinical use by mining FAERS reports of nervous system disorders AEs related to BTK inhibitors.

## Data and methods

### Data source and processing

In this study, we selected the following drugs: acalabrutinib, ibrutinib, and zanubrutinib. FAERS data from the first quarter of 2013 through the fourth quarter of 2024 were collected, with inclusion criteria requiring that BTK inhibitors be listed as the primary suspect (PS) drug in the report. Data extraction and preprocessing were conducted using R software (version 4.3.1). The initial dataset contained 22,375,298 reports. After excluding 3,749,303 duplicate records according to FDA guidelines, key information such as PRIMARYID, AGE, and GENDER was extracted. For reports sharing the same case ID, only the entry with the most recent FDA_DATE was retained; if both CASEID and FDA_DATE were identical, the record with the highest PRIMARYID was kept. AEs were coded using the Preferred Terms (PT) of MedDRA (version 27.1) and categorized by System Organ Class (SOC). Finally, all PTs categorized under the “Nervous system disorders” SOC were included (Figure 1).

**FIGURE 1:**
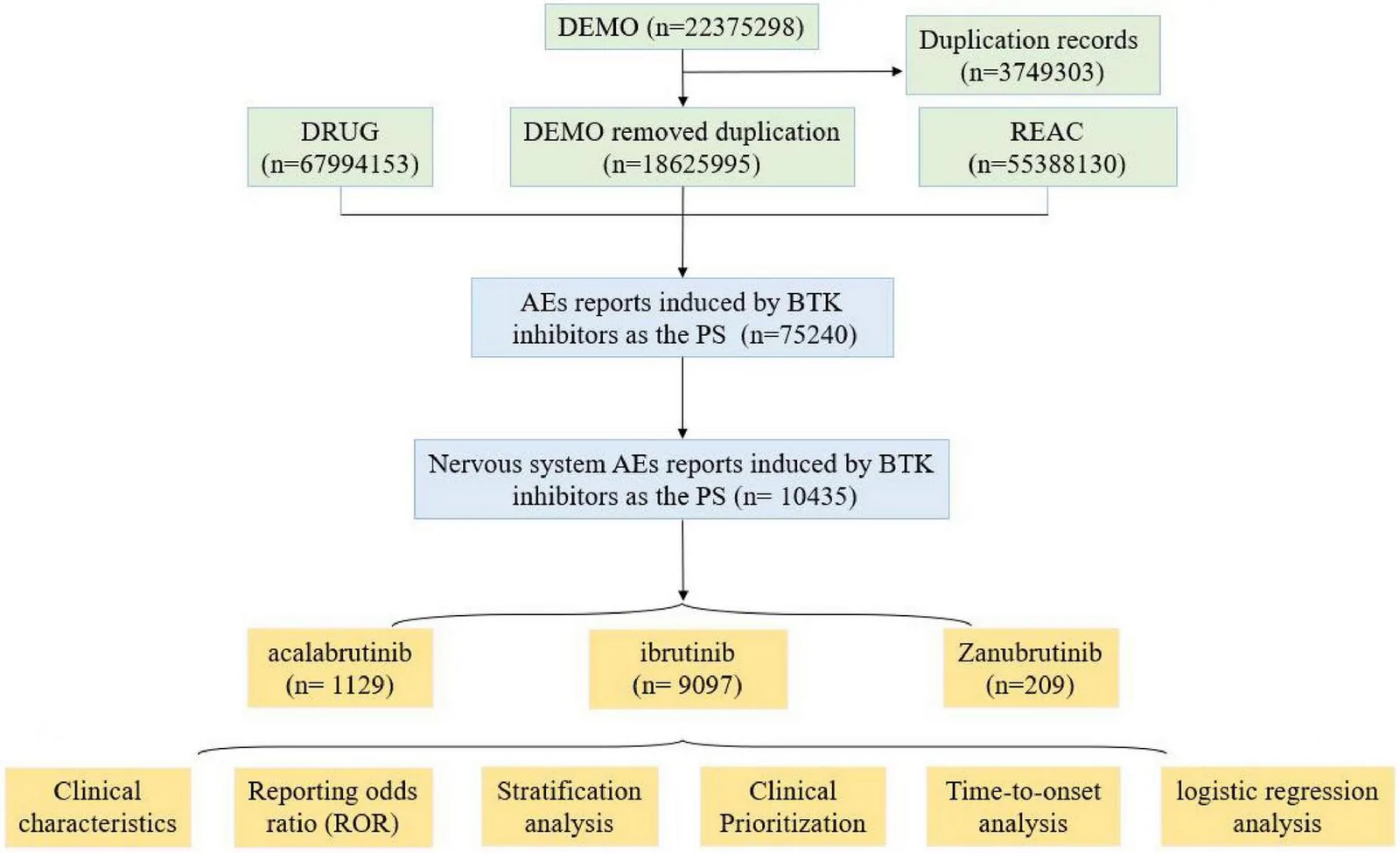
FAERS database’s pipeline flowchart for screening BTK inhibitor-associated nervous system disorder AEs.

### Statistical analysis

To investigate potential associations between BTK inhibitors and nervous system disorders AEs, disproportionality analysis was employed. This pharmacovigilance tool uses a 2 × 2 contingency table to compare the observed frequency of AEs in exposed versus unexposed populations (Supplementary Table 1). Two disproportionality methods were used to detect drug–AE signals: reporting odds ratio (ROR) and proportional reporting ratio (PRR). ROR can adjust for bias and is particularly suitable for low event counts (12), while PRR has higher specificity (13). The calculation formulas and threshold criteria for these methods are shown in Supplementary Table 2, and all statistical analyses were performed using R software. Higher values indicate stronger signal intensity, implying a more significant association between the drug and the adverse event.

The time to onset of nervous system disorders AEs was calculated using the formula: onset time = adverse event date–BTK inhibitor start date. Records with data entry errors (EVENT_DT earlier than START_DT) or inaccurate dates were excluded. Median values were used to describe time to onset.

To further explore factors associated with neurological AE reporting, we fitted a multivariable logistic regression model in which the dependent variable was whether a BTK inhibitor primary-suspect report was coded to the MedDRA SOC “Nervous system disorders” (target = 1) or to other SOC categories (non-target = 0). Independent variables included age category, sex, weight category, drug, and time-to-onset where available.

Concomitant medications, underlying disease indications, and prior therapies were not included as model covariates because these variables are incompletely and heterogeneously captured in FAERS. Their potential confounding effects are therefore addressed in the Limitations and Discussion sections.

## Results

### Nervous system disorders AEs of BTK inhibitors in FAERS (2013–2024)

We first investigated the number of nervous system disorder AEs in patients receiving BTK inhibitors from Q1 2013 through Q4 2024. A total of 18,625,995 AE reports were included from the FAERS database. After excluding duplicate reports, 75,240 cases of AEs with BTK inhibitors were identified, of which 10,435 were nervous system disorders AEs, comprising a substantial proportion. The frequency of nervous system disorder AEs varied among BTK inhibitors. These cases were associated with acalabrutinib (*n* = 1,129), ibrutinib (*n* = 9,097), and zanubrutinib (*n* = 209) (Figure 2). The year-of-report trend indicates that the number of nervous system disorders AE cases has increased significantly in recent years (Figure 3).

**FIGURE 2:**
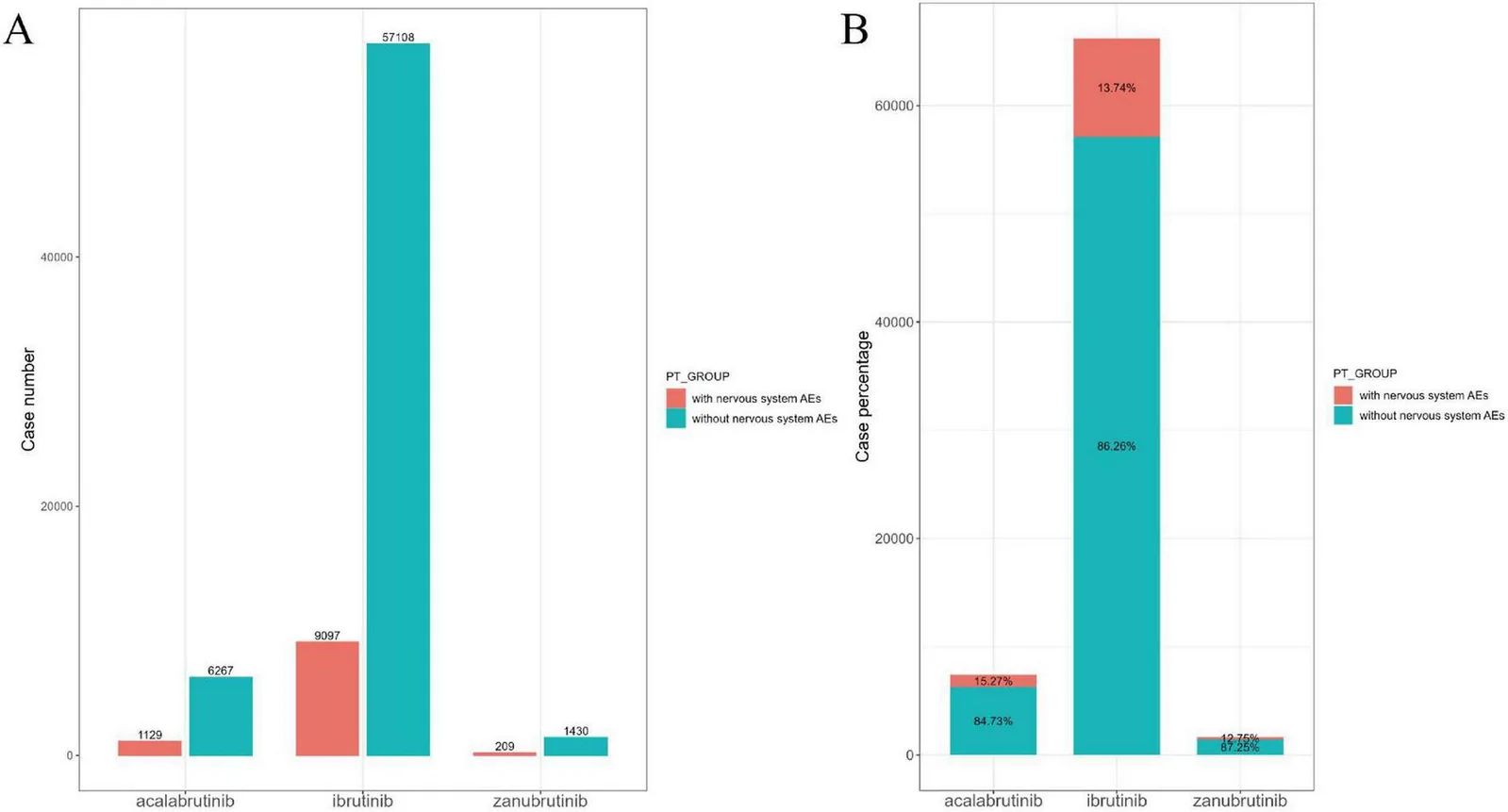
**(A)** Bar chart shows the number of reported nervous system disorder AEs of different BTK inhibitors in the FAERS database from 2013 Q1 to 2024 Q4. **(B)** Proportional bar chart shows the percentage of different BTK inhibitors and nervous system disorder events in the FAERS database from 2013 Q1 to 2024 Q4.

**FIGURE 3:**
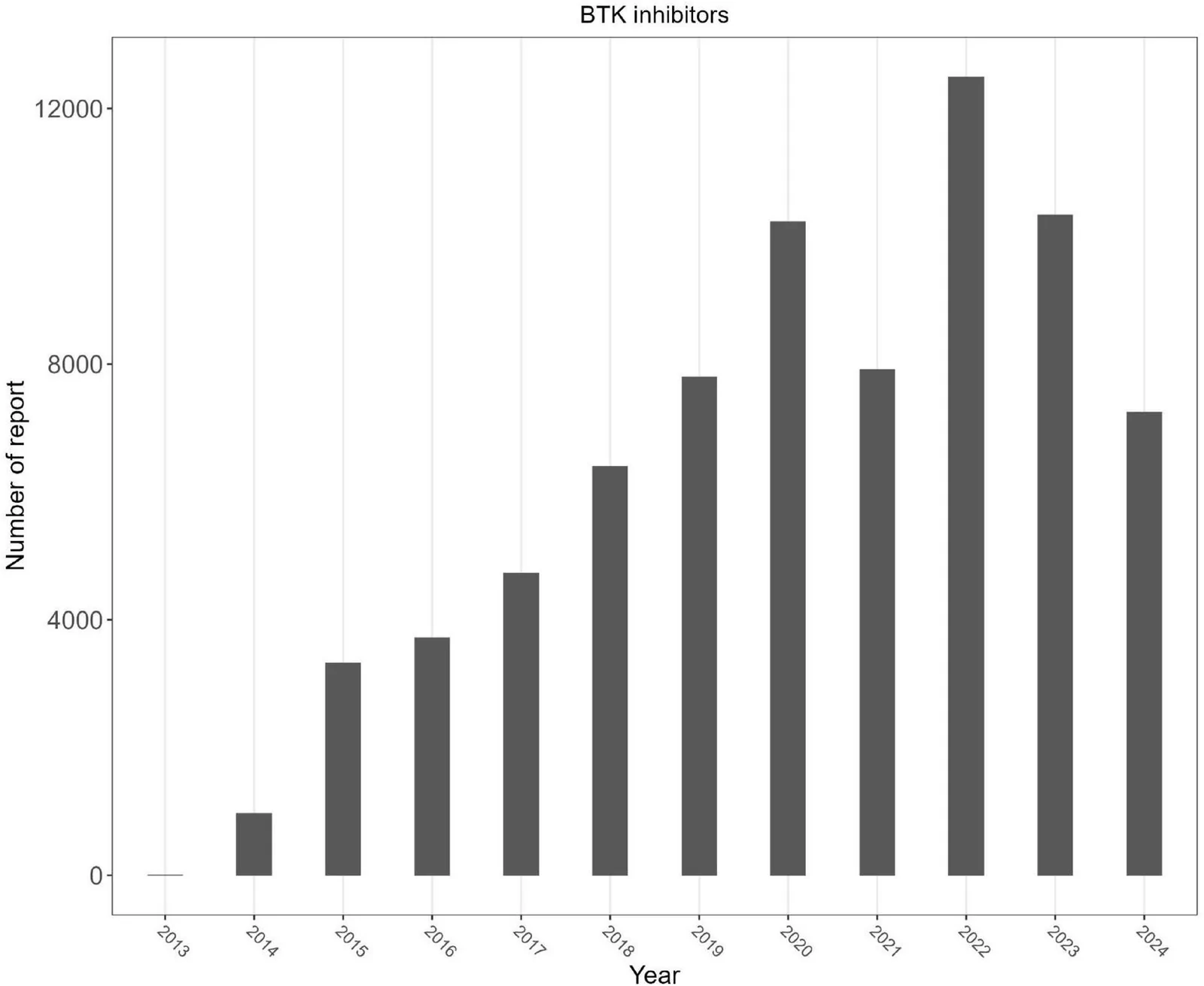
Report counts with BTK inhibitor-associated nervous system disorder AEs yearly from 2013 Q1 to 2024 Q4.

### Clinical characteristics of BTK inhibitor–related nervous system disorder AEs

A total of 10,435 nervous system disorders AE cases attributed to BTK inhibitors (as PS) were extracted from the FAERS, along with patients’ clinical characteristics. 53.04% of cases were male, 37.99% were female, and sex information was unavailable for 9.97%. The largest age group was 65–85 years old (34.14%). Weight data were missing in 81.55% of cases. Consumers submitted the majority of reports (68.78%). By country of report, the United States was the most common, accounting for 75.30% of cases (Table 1).

**TABLE 1:** Characteristics of nervous system disorders AEs correlated with BTK inhibitors.

Characteristics	Acalabrutinib	Ibrutinib	Zanubrutinib
Gender			
Female	341 (30.2%)	3620 (39.8%)	3 (1.4%)
Male	462 (40.9%)	5071 (55.7%)	2 (1.0%)
Missing	326 (28.9%)	406 (4.5%)	204 (97.6%)
Age			
<18	0 (0.0%)	45 (0.5%)	0 (0.0%)
18–65	31 (2.7%)	1207 (13.3%)	1 (0.5%)
65–85	80 (7.1%)	3481 (38.3%)	2 (1.0%)
>85	231 (20.5%)	435 (4.8%)	1 (0.5%)
Missing	787 (69.7%)	3929 (43.2%)	205 (98.1%)
Weight (kg)			
<50	13 (1.2%)	140 (1.5%)	0 (0%)
50–100	47 (4.2%)	223 (2.5%)	0 (0%)
>100	213 (18.9%)	1,285 (14.1%)	4 (1.9%)
Missing	856 (75.8%)	7,449 (81.9%)	205 (98.1%)
Reporters			
Consumer	745 (66.0%)	6,309 (69.4%)	123 (58.9%)
Physician	167 (14.8%)	1,473 (16.2%)	52 (24.9%)
Pharmacist	26 (2.3%)	177 (1.9%)	14 (6.7%)
Unknown	134 (11.9%)	17 (0.2%)	1 (0.5%)
Reporting country			
The United States	957 (84.8%)	6,744 (74.1%)	157 (75.1%)
Canada	44 (3.9%)	256 (2.8%)	12 (5.7%)
Great Britain	18 (1.6%)	156 (1.7%)	3 (1.4%)

### Disproportionality analysis of BTK inhibitor–related nervous system disorders AEs

The disproportionality analysis results are summarized in Table 2. None of the three BTK inhibitors showed a positive overall disproportionality signal for reports coded to the MedDRA SOC “Nervous system disorders”: acalabrutinib had an ROR of 0.95 (95% CI 0.90–1.01), ibrutinib had an ROR of 0.68 (95% CI 0.67–0.69), and zanubrutinib had an ROR of 0.61 (95% CI 0.54–0.69). These findings do not support an overall SOC-level increase in disproportionate reporting for neurological AEs. Importantly, the larger absolute number of neurological AE reports for ibrutinib should not be interpreted as a stronger SOC-level signal. Positive disproportionality signals were instead observed at the PT level for selected neurological events.

**TABLE 2:** Safety adverse events among different BTK inhibitors.

Drug	BTK inhibitor associated AEs	BTK inhibitor-associated nervous system AEs	BTK inhibitor-associated nervous system AEs as PS	ROR (95%Cl)	PRR (XX)
Acalabrutinib	17,511	1,422	1,129	0.95 (0.9–1.01)	0.96 (2.85)
Ibrutinib	206,270	12,234	9,097	0.68 (0.67–0.69)	0.7 (1728.79)
Zanubrutinib	5,153	275	209	0.61 (0.54–0.69)	0.63 (65.48)

ROR, reporting odds ratio; PRR, proportional reporting ratio; CI, confidence interval; AEs, adverse events.

At the PT level, 212 positive signals were identified. The statistically significant PT-level signals for acalabrutinib, ibrutinib, and zanubrutinib involved 11 signals (ROR range: 2.04–10.84), 18 signals (ROR range: 2.04–16.64), and 3 signals (ROR range: 2.31–4.43), respectively (Supplementary Tables 3–5).

Using high-level group term (HLGT) classification, PT-level AEs were grouped into higher-level categories corresponding to nervous system disorders. The number of HLGT-level nervous system disorders AEs for acalabrutinib, ibrutinib, and zanubrutinib was 46, 45, and 30, respectively (Figure 4). The HLGT signals for acalabrutinib included migraine headaches, multiple sclerosis (acute and progressive), Parkinson’s disease and Parkinsonism, narcolepsy and hypersomnia, coordination and balance disturbances, and optic nerve disorders NEC, etc. The HLGT signals for ibrutinib included acute polyneuropathies, encephalopathies NEC, multiple sclerosis (acute and progressive), speech and language abnormalities, dyskinesias and movement disorders NEC, and spinal cord and nerve root disorders NEC et al. The HLGT signals for zanubrutinib included speech and language abnormalities, narcolepsy and hypersomnia, dyskinesias and movement disorders NEC, paralysis and paresis (excluding cranial nerve), spinal cord and nerve root disorders NEC, and optic nerve disorders NEC etc.

**FIGURE 4:**
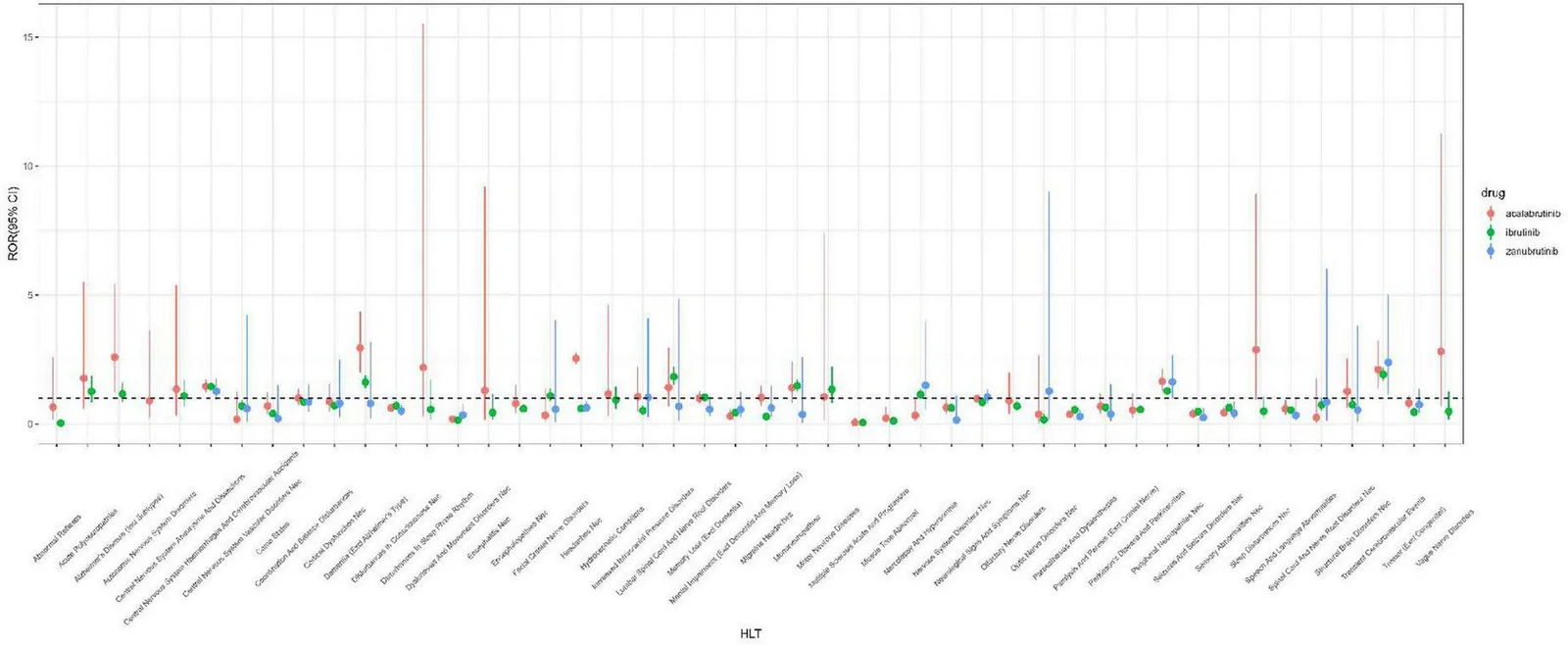
Forest plots of AEs under HLGT classification of different BTK inhibitors. HLGT, high-level group term.

To characterize the clinical spectrum of frequently reported neurological AEs, we summarized the 50 most commonly reported PTs together with their disproportionality metrics. Frequency and disproportionality should be interpreted separately. The most frequently reported PTs were headache, dizziness, cerebrovascular accident, loss of consciousness, and peripheral neuropathy. Among these commonly reported PTs, positive disproportionality signals were observed for selected events such as cerebrovascular accident, loss of consciousness, memory impairment, peripheral neuropathy, cerebral hemorrhage, balance disorder, transient ischaemic attack, dementia, intracranial hemorrhage, subarachnoid hemorrhage, haemorrhagic stroke, postural dizziness, and brain fog, whereas other frequent PTs such as headache and dizziness did not show positive disproportionality (Figure 5 and Table 3).

**FIGURE 5:**
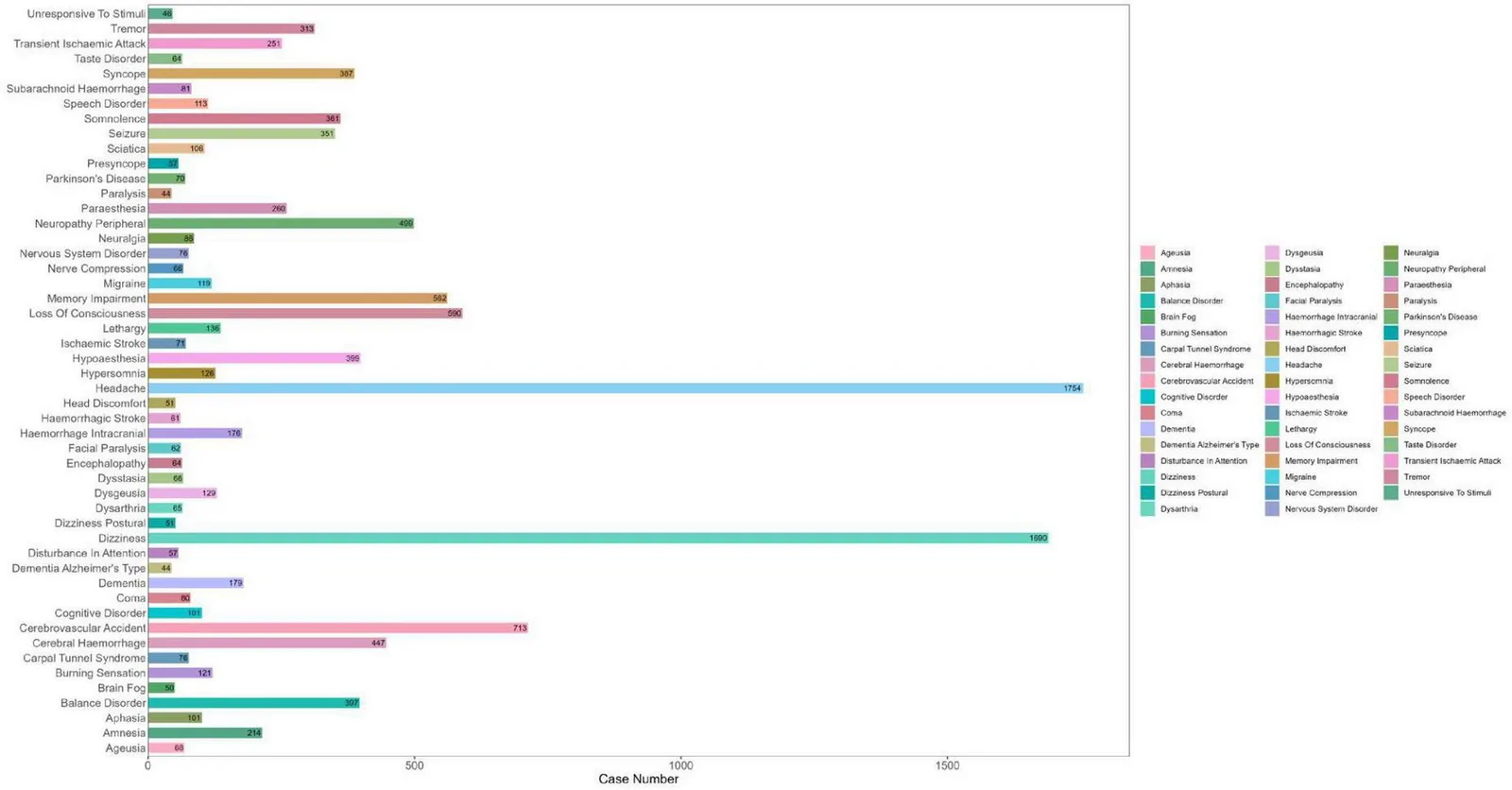
The number of reported cases of the first 50 types of BTK inhibitors related to nervous system disorders AEs. PT, preferred term.

**TABLE 3:** Signal values of the first 50 types of BTK inhibitors related to nervous system disorders AEs.

PT	Case number	ROR (95%Cl)	PRR (XX)
Headache	1,754	0.75 (0.72–0.79)	0.75 (142.9)
Dizziness	1,690	0.91 (0.87–0.96)	0.91 (13.79)
Cerebrovascular accident	713	1.1 (1.02–1.18)	1.1 (6.45)
Loss of consciousness	590	1.23 (1.13–1.33)	1.23 (25.25)
Memory impairment	562	1.09 (1.01–1.19)	1.09 (4.42)
Neuropathy peripheral	499	1.45 (1.33–1.58)	1.45 (69.01)
Cerebral hemorrhage	447	3.36 (3.06–3.69)	3.35 (728.63)
Hypoesthesia	399	0.7 (0.64–0.78)	0.7 (49.59)
Balance disorder	397	1.22 (1.1–1.35)	1.22 (15.48)
Syncope	387	1.02 (0.92–1.13)	1.02 (0.13)
Somnolence	361	0.48 (0.43–0.53)	0.48 (200.89)
Seizure	351	0.54 (0.49–0.6)	0.54 (133.71)
Tremor	313	0.5 (0.45–0.56)	0.5 (158.01)
Paraesthesia	260	0.43 (0.38–0.49)	0.44 (190.91)
Transient ischemic attack	251	1.94 (1.72–2.2)	1.94 (113.77)
Amnesia	214	0.86 (0.75–0.98)	0.86 (4.95)
Dementia	179	1.81 (1.56–2.09)	1.81 (64.02)
Hemorrhage intracranial	176	3.04 (2.62–3.53)	3.04 (237.66)
Lethargy	136	0.63 (0.53–0.74)	0.63 (30.24)
Dysgeusia	129	0.45 (0.38–0.53)	0.45 (88.27)
Hypersomnia	126	1.19 (1–1.42)	1.19 (3.88)
Burning sensation	121	0.46 (0.38–0.55)	0.46 (77.12)
Migraine	119	0.35 (0.29–0.41)	0.35 (147.19)
Speech disorder	113	0.57 (0.47–0.69)	0.57 (36.46)
Sciatica	106	1.89 (1.56–2.28)	1.89 (43.74)
Aphasia	101	0.87 (0.71–1.06)	0.87 (1.99)
Cognitive disorder	101	0.59 (0.48–0.71)	0.59 (29.47)
Neuralgia	86	0.97 (0.78–1.2)	0.97 (0.09)
Subarachnoid hemorrhage	81	2.1 (1.69–2.61)	2.1 (46.14)
Coma	80	0.44 (0.36–0.55)	0.44 (55.39)
Nervous system disorder	76	0.63 (0.5–0.79)	0.63 (16.58)
Carpal tunnel syndrome	76	1.46 (1.16–1.83)	1.46 (10.84)
Ischemic stroke	71	1.05 (0.84–1.33)	1.05 (0.2)
Parkinson’s disease	70	1.01 (0.8–1.28)	1.01 (0.01)
Ageusia	68	0.73 (0.57–0.92)	0.73 (6.9)
Dysstasia	66	0.59 (0.47–0.76)	0.59 (18.28)
Nerve compression	66	1.96 (1.54–2.49)	1.96 (30.58)
Dysarthria	65	0.46 (0.36–0.58)	0.46 (41.86)
Taste disorder	64	1 (0.78–1.28)	1 (0)
Encephalopathy	64	0.7 (0.55–0.9)	0.7 (7.88)
Facial paralysis	62	1.16 (0.9–1.48)	1.16 (1.31)
Hemorrhagic stroke	61	2.03 (1.58–2.62)	2.03 (31.81)
Presyncope	57	0.64 (0.49–0.83)	0.64 (11.55)
Disturbance in attention	57	0.27 (0.21–0.36)	0.27 (109.26)
Head discomfort	51	0.8 (0.61–1.06)	0.8 (2.46)
Dizziness postural	51	1.52 (1.16–2)	1.52 (9.06)
Brain fog	50	1.71 (1.29–2.26)	1.71 (14.57)
Unresponsive to stimuli	46	0.47 (0.35–0.63)	0.47 (26.79)
Paralysis	44	0.8 (0.59–1.07)	0.8 (2.3)
Dementia Alzheimer’s type	44	1.26 (0.94–1.7)	1.26 (2.37)

ROR, reporting odds ratio; PRR, proportional reporting ratio; CI, confidence interval; AEs, adverse events; PT, preferred term.

To prioritize clinically serious neurological AE reports, we calculated the proportion of reports with a fatal outcome among selected PTs. Cerebrovascular accident and cerebral hemorrhage showed the highest reported fatal outcome proportions, exceeding 15%. However, FAERS does not allow adjudication of whether death was caused by the neurological event itself, the underlying malignancy, disease progression, comorbid conditions, or co-administered therapies. These results should therefore be interpreted as fatal outcome proportions among reports containing the corresponding PTs, rather than event-specific mortality rates (Figure 6).

**FIGURE 6:**
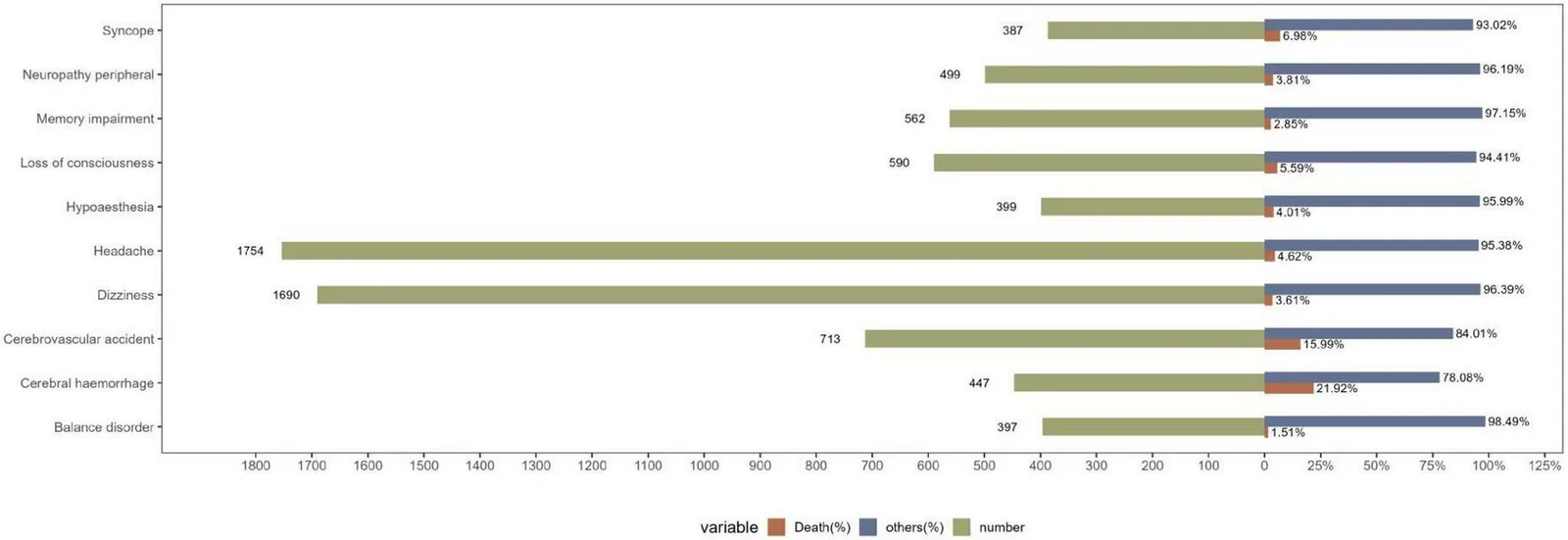
Death cases and their proportion in BTK inhibitors concomitantly with nervous system disorders AEs.

### Time to onset of nervous system disorders AEs

Figure 7 depicts the time to onset of nervous system disorders AEs for each BTK inhibitor. The median time to onset after starting acalabrutinib was 27 days (IQR: 8–133 days). In contrast, the onset time for ibrutinib was the longest, at 123 days (IQR: 21–530 days). For zanubrutinib, the median time was 45 days (IQR: 10–146 days). This analysis reveals the onset timing of nervous system disorder AEs induced by BTK inhibitors, providing new insights into their clinical use and potential neurotoxicity.

**FIGURE 7:**
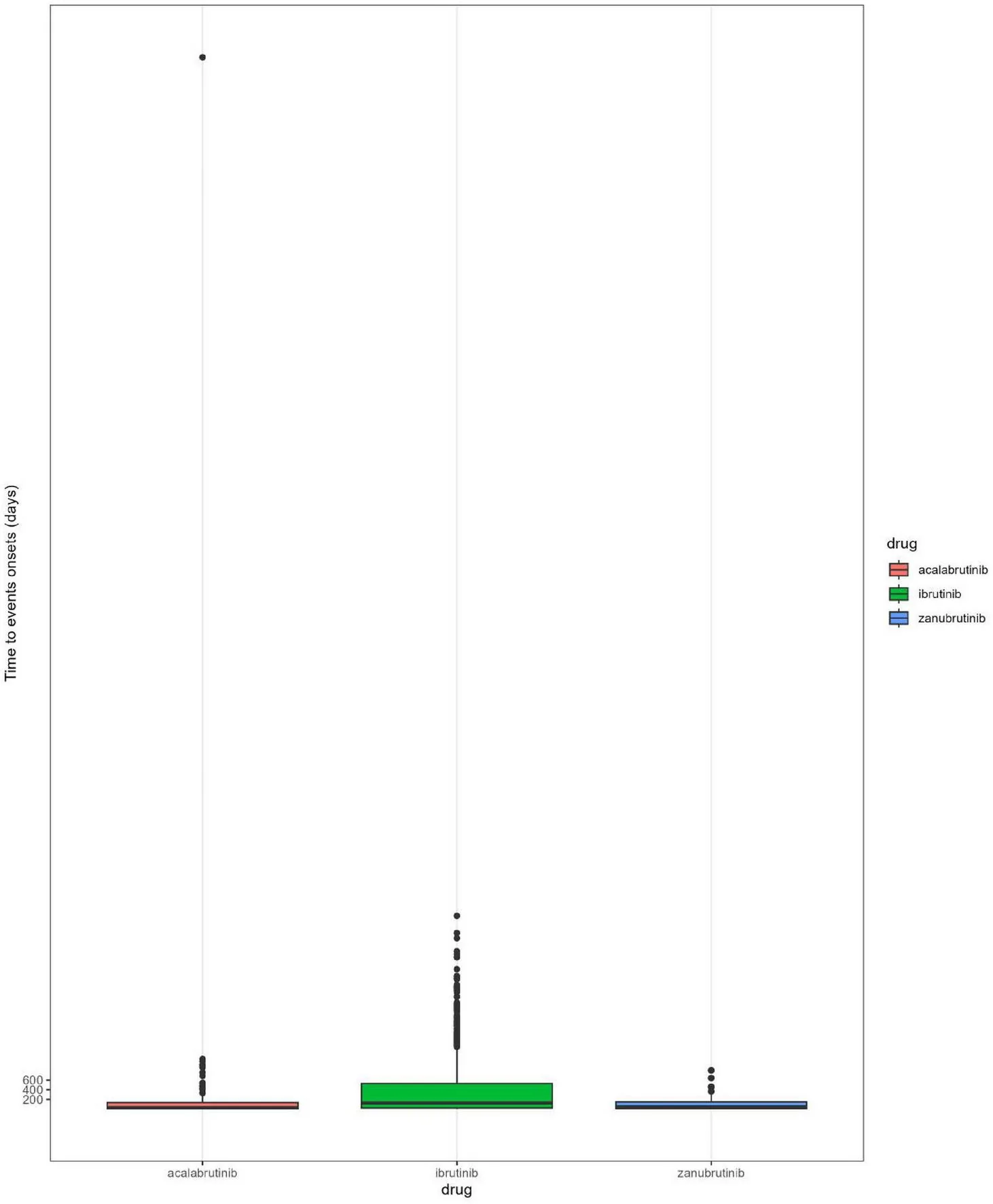
Time to the onset of nervous system disorders AEs.

### Severe versus non-severe nervous system disorder AEs

As shown in Supplementary Table 6, statistically significant differences were observed in PTs between severe and non-severe cases of BTK inhibitor-associated nervous system disorders AEs. Twenty-three AEs were significantly more likely to be reported as serious events (*p* < 0.05), for example, tremor (χ^2^ = 4.53, *p* < 0.05), carpal tunnel syndrome (χ^2^ = 5.00, *p* < 0.05), spinal cord hemorrhage (χ^2^ = 7.26, *p* < 0.05), cognitive disorder (χ^2^ = 5.47, *p* < 0.05), burning sensation (χ^2^ = 5.58, *p* < 0.05), dementia (χ^2^ = 5.87, *p* < 0.05), cerebral ischemia (χ^2^ = 8.44, *p* < 0.05), loss of consciousness (χ^2^ = 6.36, *p* < 0.05), etc. The remaining 114 AEs were more likely to be reported as non-serious (*p* > 0.05), for example lethargy (χ^2^ = 4.15, *p* > 0.05), ataxia (χ^2^ = 5.05, *p* > 0.05), nerve compression (χ^2^ = 4.09, *p* > 0.05), aphasia (χ^2^ = 3.92, *p* > 0.05), brain stem hemorrhage (χ^2^ = 6.48, *p* > 0.05), and speech disorder (χ^2^ = 3.51, *p* > 0.05), etc.

### Logistic regression analysis

Multivariable logistic regression was performed to explore factors associated with neurological AE reporting among BTK inhibitor primary-suspect reports (Figure 8). In this model, reports coded to the MedDRA SOC “Nervous system disorders” were defined as target events (target = 1), whereas reports coded to other SOCs were defined as non-target events (non-target = 0).

**FIGURE 8:**
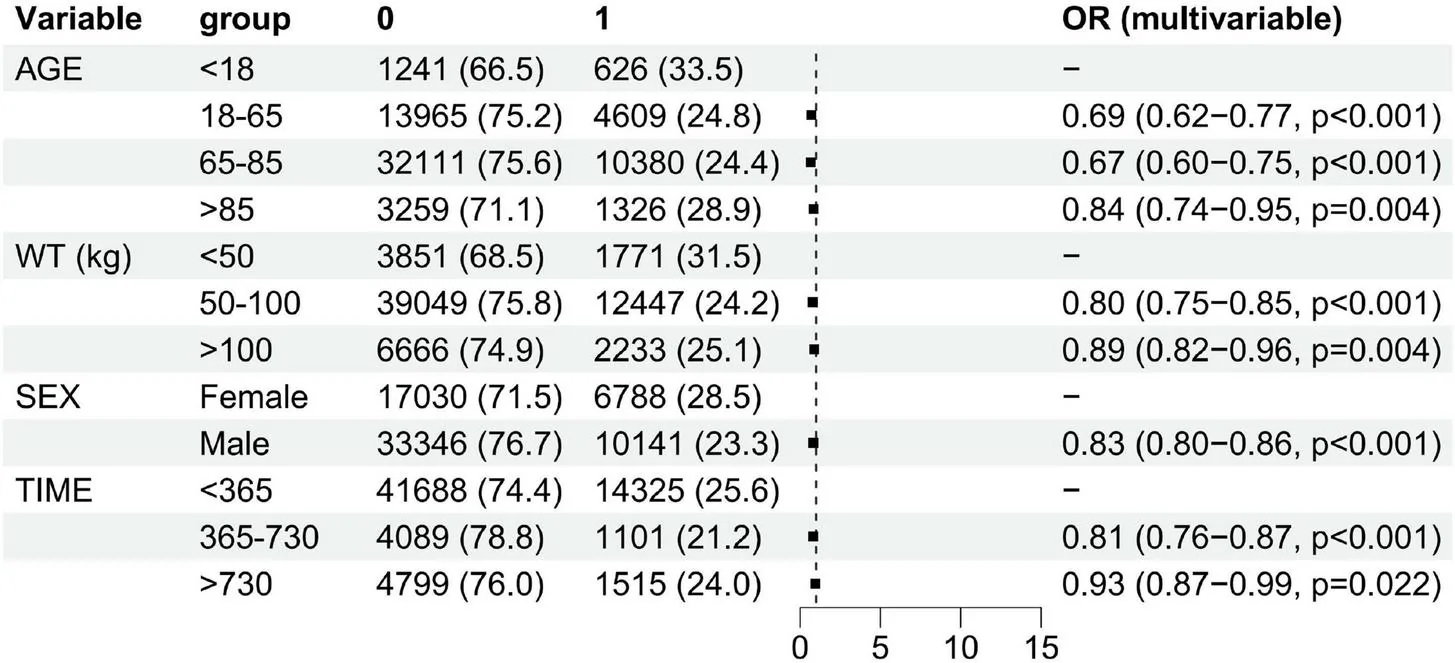
Multivariate logistic regression results of nervous system disorder AEs with different factors. Target = 1: neurological AE reports; non-target = 0: BTK inhibitor reports coded to other SOC categories.

Compared with patients aged < 18 years, adult age groups showed lower odds of neurological AE reporting, including those aged 18–65 years (OR = 0.69, 95% CI: 0.62–0.77, *p* < 0.001), 65–85 years (OR = 0.67, 95% CI: 0.60–0.75, *p* < 0.001), and > 85 years (OR = 0.84, 95% CI: 0.74–0.95, *p* = 0.004). For body weight, patients weighing 50–100 kg (OR = 0.80, 95% CI: 0.75–0.85, *p* < 0.001) and > 100 kg (OR = 0.89, 95% CI: 0.82–0.96, *p* = 0.004) had lower odds of neurological AE reporting compared with those weighing < 50 kg. Male sex was also associated with lower odds of neurological AE reporting compared with female sex (OR = 0.83, 95% CI: 0.80–0.86, *p* < 0.001).

Regarding time to onset, reports with onset times of 365–730 days (OR = 0.81, 95% CI: 0.76–0.87, *p* < 0.001) and > 730 days (OR = 0.93, 95% CI: 0.87–0.99, *p* = 0.022) showed lower odds of neurological AE reporting compared with reports with onset times < 365 days. These findings suggest that neurological AE reporting was relatively more frequent among reports from younger patients, female patients, patients with lower body weight, and reports with earlier onset times. However, these associations should be interpreted cautiously because FAERS contains substantial missing demographic and clinical information and cannot establish causal or incidence relationships.

Because of sparse reporting and extensive missing covariate data for zanubrutinib, the adjusted multivariable logistic regression model was restricted to acalabrutinib and ibrutinib primary-suspect reports.

## Discussion

BTK inhibitors have transformed the treatment of B-cell malignancies (14–16). But their neurological safety profiles remain incompletely characterized in routine practice (5, 17). In this large-scale, nervous-system-focused pharmacovigilance analysis, we identified heterogeneous reporting patterns across BTK inhibitors and across neurological PTs. These findings should be interpreted as pharmacovigilance signals derived from spontaneous reports, not as proof of causality or incidence differences.

### BTK inhibitor-associated neurological toxicity profile

Our comprehensive analysis identified distinct neurological safety profiles among BTK inhibitors, with significant variability in both the frequency and severity of AEs. The most frequently reported nervous system disorder AEs included cerebrovascular accident (mortality rate: 15.99%), cerebral hemorrhage (mortality rate: 21.92%), syncope (6.98%), peripheral neuropathy (3.81%), memory impairment (2.85%), loss of consciousness (5.59%), hypoesthesia (4.01%), headache (4.62%), dizziness (3.61%), and balance disorder (1.15%). These findings underscore the heterogeneous nature of BTK inhibitor–induced neurotoxicity and highlight the critical importance of neurological monitoring in patients receiving these therapies.

### Mechanisms underlying BTK inhibitor neurological toxicity

The observed pattern of nervous system disorder AEs reflects the complex pharmacological properties of BTK inhibitors and their differential target engagement profiles. First-generation agents like ibrutinib demonstrate broader kinase inhibition patterns, affecting multiple off-target proteins, including phospholipase C-gamma, protein kinase C, and various components of the PI3K–Akt pathway (18–20). Off-target kinase inhibition contributes to increased toxicity compared to more selective second-generation inhibitors such as acalabrutinib and zanubrutinib (21, 22). However, because FAERS lacks adjudicated clinical detail and exposure denominators, these mechanistic considerations are hypothesis-generating and should not be interpreted as established explanations for the reported events.

### Central nervous system penetration and direct effects

The neurological toxicity observed with BTK inhibitors likely stems from multiple overlapping mechanisms. A fundamental consideration is the blood–brain barrier penetration of these agents. While traditional BTK inhibitors like ibrutinib, acalabrutinib, and zanubrutinib have limited CNS penetration (brain-to-plasma ratios typically < 10%), even minimal CNS exposure may be sufficient to engage neurologically relevant targets (23, 24). Recent development of CNS-penetrant BTK inhibitors such as tolebrutinib and fenebrutinib, designed specifically for neurological indications, has demonstrated that enhanced brain penetration is achievable and may be associated with different toxicity profiles (25, 26). Direct clinical evidence for meaningful CNS exposure of acalabrutinib and zanubrutinib remains limited, and the present FAERS analysis cannot determine whether any reported CNS events were mediated by direct CNS drug exposure. Discussion of BBB penetration is therefore best regarded as a plausible, but unconfirmed, mechanistic hypothesis.

The direct effects of BTK inhibition within the CNS involve multiple cellular targets, including microglia, astrocytes, and neurons (27). BTK is expressed in CNS-resident immune cells, where it regulates inflammatory signaling pathways (5, 28). Inhibition of microglial BTK signaling can modulate neuroinflammatory responses through effects on NLRP3 inflammasome activation, cytokine production, and phagocytic function (29, 30). While this may be therapeutically beneficial in certain neuroinflammatory conditions, it could also compromise normal CNS homeostasis and neuronal protection mechanisms.

### Vascular and haemostatic mechanisms

The increased incidence of cerebrovascular events, including stroke and intracerebral hemorrhage, represents one of the most serious neurological complications of BTK inhibitor therapy (31, 32). BTK plays critical roles in platelet function and haemostasis through its effects on glycoprotein VI signaling and platelet aggregation (33, 34). Inhibition of BTK-dependent platelet activation increases bleeding risk, particularly in the setting of concurrent anticoagulation therapy or underlying vascular pathology (35). The combination of anticoagulation requirements for atrial fibrillation management and BTK inhibitor–induced platelet dysfunction creates a complex clinical scenario requiring careful risk–benefit assessment (36). Additionally, BTK inhibitors may exert direct effects on the cerebrovascular endothelium through modulation of inflammatory signaling pathways. Endothelial BTK activation regulates vascular permeability, inflammatory cell recruitment, and thrombotic responses. Disruption of these pathways could contribute to both haemorrhagic and ischaemic cerebrovascular complications through distinct mechanisms (37).

### Off-target kinase effects

The neurological toxicity profile of BTK inhibitors is significantly influenced by their selectivity profiles and off-target kinase interactions (38). Ibrutinib, as a first-generation agent, inhibits numerous kinases, including members of the EGFR family, ITK, TEC, and components of calcium signaling pathways (39). These off-target effects contribute to its broader toxicity profile, including neurological complications such as peripheral neuropathy and cognitive dysfunction (40). The inhibition of calcium signaling pathways is particularly relevant to neurological function, as these pathways are essential for synaptic transmission, neuronal excitability, and neurotransmitter release (41). Second-generation BTK inhibitors such as acalabrutinib and zanubrutinib were designed with improved selectivity profiles to reduce off-target toxicity (42, 43). However, our analysis demonstrates that these agents still exhibit significant nervous system disorders AE signals, suggesting that both BTK-dependent and independent mechanisms contribute to neurotoxicity. The persistence of certain nervous system disorder AEs across different BTK inhibitors with varying selectivity profiles indicates that some effects may be class-related rather than compound-specific.

### Time course and clinical patterns of nervous system disorders AEs

The temporal patterns of BTK inhibitor-associated nervous system disorder AEs provide important insights into their underlying mechanisms and clinical management strategies. Our analysis revealed significant variation in onset timing, with some AEs appearing early in therapy while others develop after prolonged exposure. Acute nervous system disorders AEs such as headache and dizziness typically manifest within days to weeks of treatment initiation, consistent with direct pharmacological effects on neuronal or vascular function (44). In contrast, more serious complications such as cerebrovascular events and cognitive dysfunction often develop after months to years of therapy, suggesting cumulative toxicity mechanisms or progressive pathological processes. This delayed onset pattern is particularly concerning for patients receiving continuous BTK inhibitor therapy, as it necessitates long-term neurological monitoring even in patients who initially tolerate treatment well.

The observation that certain nervous system disorder AEs, such as peripheral neuropathy, may be partially reversible upon drug discontinuation suggests that some toxic effects are related to ongoing drug exposure rather than irreversible tissue damage. This reversibility provides important clinical guidance for management decisions and supports the potential for dose modifications or drug holidays in patients experiencing neurological toxicity.

### Comparative safety profiles among BTK inhibitors

The differential neurological safety profiles observed among BTK inhibitors provide important clinical guidance for therapeutic selection and patient counseling. Ibrutinib, as the first-generation agent, demonstrates the highest overall incidence of nervous system disorder AEs and the broadest spectrum of complications. The agent’s promiscuous kinase inhibition profile contributes to effects ranging from mild headache and dizziness to severe complications such as stroke and progressive multifocal leukoencephalopathy.

Acalabrutinib exhibits a distinct neurological toxicity pattern characterized by frequent but generally mild headache (22–51% incidence) with minimal long-term sequelae (45). The agent’s improved selectivity profile is associated with a reduced incidence of severe neurological complications compared to ibrutinib (46), although significant AEs such as reversible posterior leukoencephalopathy syndrome have been reported (47). The shorter half-life and improved BTK occupancy profile of acalabrutinib may contribute to its more favorable neurological tolerability (48).

The comparative interpretation of neurological safety across BTK inhibitors requires caution. Ibrutinib contributed the largest number and broadest spectrum of reported neurological AEs in FAERS, which may reflect its longer market availability, broader clinical use, and less selective kinase profile. This should not be interpreted as evidence of a stronger SOC-level neurological disproportionality signal, because no overall positive SOC-level signal was observed for any of the three agents. Acalabrutinib and zanubrutinib were developed to improve selectivity and overall tolerability relative to ibrutinib, and comparative clinical evidence in CLL has suggested improved tolerability for zanubrutinib while maintaining efficacy (49, 50). Nevertheless, our FAERS-based comparisons remain indirect and are susceptible to reporting bias, confounding, and unequal exposure across agents, particularly given the relatively small number of zanubrutinib reports.

### Clinical implications and management strategies

The identification of significant nervous system disorders AE signals associated with BTK inhibitors necessitates comprehensive clinical management strategies that address both prevention and early detection of complications. Pretreatment neurological assessment should include evaluation of baseline cognitive function, a thorough neurological examination, and identification of risk factors for cerebrovascular disease. Patients with pre-existing neurological conditions, a history of stroke, or significant bleeding risk factors require particularly careful evaluation and monitoring. During therapy, regular neurological monitoring should include assessment for new or worsening headache, cognitive changes, motor or sensory symptoms, and signs of cerebrovascular complications. The high mortality rates associated with certain nervous system disorder AEs, particularly cerebrovascular accidents and CNS hemorrhage, underscore the importance of prompt recognition and intervention.

The management of BTK inhibitor-associated nervous system disorder AEs requires individualized approaches based on the severity and nature of the complications. Mild symptoms such as headache may be managed symptomatically while continuing therapy, whereas severe complications such as stroke or CNS hemorrhage typically require immediate drug discontinuation and specialized neurological care. The decision to resume BTK inhibitor therapy after a neurological AE should carefully weigh the risks and benefits, considering the availability of alternative treatments and the underlying malignancy status of the patient.

### Pharmacovigilance implications and future directions

This analysis demonstrates the critical importance of post-marketing surveillance in identifying neurological safety signals that may not be apparent in controlled clinical trials. The FAERS database analysis revealed numerous nervous system disorder AEs that were either not reported or under-represented in pivotal clinical studies, highlighting limitations in pre-approval safety assessment. The identification of rare but serious complications such as aseptic meningitis and progressive multifocal leukoencephalopathy through real-world data emphasizes the value of comprehensive pharmacovigilance programs.

Future research directions should focus on several key areas, including the development of predictive biomarkers for neurological toxicity, investigation of neuroprotective strategies, and optimisation of patient selection criteria. The emerging understanding of BTK’s role in neuroinflammation and CNS homeostasis suggests potential opportunities for developing CNS-selective BTK inhibitors with improved therapeutic windows. Additionally, investigation of combination strategies that might mitigate neurological toxicity while preserving therapeutic efficacy represents an important research priority.

The development of standardized neurological monitoring protocols and toxicity grading systems specific to BTK inhibitors would facilitate improved clinical management and research efforts. Collaborative initiatives between hematology–oncology and neurology specialities are essential for advancing understanding of BTK inhibitor neurotoxicity and developing optimal management strategies.

## Limitations

Several limitations should be emphasized. First, FAERS is a spontaneous reporting system subject to under-reporting, stimulated reporting, duplicate submissions, and variable report quality. Second, disproportionality analysis identifies reporting imbalances rather than incidence, relative risk in exposed populations, or causal relationships. Third, no overall positive SOC-level disproportionality signal was observed for the MedDRA SOC “Nervous system disorders” for any of the three BTK inhibitors; accordingly, the main findings of this study are driven by selected PT- and HLGT-level signals rather than by a class-wide SOC-level neurological signal. Fourth, important confounders—including concomitant anticoagulants, antiplatelet agents, corticosteroids, other anticancer therapies, underlying disease characteristics, and prior treatments—could not be reliably controlled because these variables are incompletely and heterogeneously recorded in FAERS. This limitation is particularly relevant to cerebrovascular and haemorrhagic events. Fifth, missing demographic and date information was substantial, especially among zanubrutinib reports, limiting the robustness of adjusted analyses and raising the possibility of selection bias in complete-case models. Sixth, absolute report counts are strongly influenced by differences in approval date, market uptake, and cumulative exposure; therefore, comparisons involving zanubrutinib should be interpreted cautiously. Seventh, FAERS does not allow reliable adjudication of whether death was attributable to the neurological event itself, the underlying malignancy, disease progression, comorbid conditions, or concomitant therapies. Given recent multidimensional pharmacovigilance analyses of BTK inhibitors, the novelty of the present work lies primarily in its nervous-system-focused characterization rather than in being the first large database study in this therapeutic area.

In addition, this study used ROR and PRR as frequentist disproportionality metrics but did not incorporate Bayesian signal-detection methods such as BCPNN or MGPS. This may limit the robustness assessment of rare or sparse neurological AE signals. Moreover, although key methodological elements were reported, including the FAERS data source, study period, deduplication strategy, primary-suspect drug definition, MedDRA version, and signal-detection criteria, the study was not prospectively designed according to READUS-PV recommendations. This limitation should be considered when interpreting the transparency and reproducibility of the findings.

## Conclusion

In this large-scale FAERS analysis, we identified distinct reporting patterns and pharmacovigilance signals of nervous system disorders AEs among BTK inhibitors, particularly at the PT and HLGT levels, rather than an overall SOC-level disproportionality signal. Cerebrovascular and haemorrhagic PTs were associated with comparatively high proportions of reported fatal outcomes, although FAERS does not permit adjudication of cause of death. These findings support careful neurological monitoring and individualized risk assessment—especially in patients with cerebrovascular risk factors or concomitant antithrombotic therapy—but they should not be interpreted as evidence of causality.

## Data Availability

The raw data supporting the conclusions of this article will be made available by the authors, without undue reservation.
