# Supplementary material for: Peripheral and central nervous system toxicities of Bruton tyrosine kinase Inhibitors: a real-world pharmacovigilance study
Source: Front Med (Lausanne). 2026 Sep 15;13:1903660. doi: 10.3389/fmed.2026.1903660 (PMC13621959; doi:10.3389/fmed.2026.1903660)
Supplement: Supplementary file 1 [file Table_1.docx]

**Supplementary Table 1** Two-by-two contingency table for disproportionality analyses.

|  | Target AEs | Other AEs | Total |
| --- | --- | --- | --- |
| Target drugs | a | b | a+b |
| Other drugs | c | d | c+d |
| Total | a+c | b+d | a+b+c+d |

Abbreviation: AEs, adverse events; a, number of reports containing both the target drug and target adverse drug reaction; b, number of reports containing other adverse drug reaction of the target drug; c, number of reports containing the target adverse drug reaction of other drugs; d, number of reports containing other drugs and other adverse drug reactions.

**Supplementary Table 2** Four major algorithms used for signal detection.

| Algorithms | Calculation Formula | Threshold |
| --- | --- | --- |
| ROR | $\begin{matrix} ROR=\frac{(a/c)}{(b/d)}=\frac{ad}{bc} \\ SE(InROR)=\sqrt{\left(\frac{1}{a}+\frac{1}{b}+\frac{1}{c}+\frac{1}{d}\right)} \\ 95\%CI=eIn(ROR)\pm1.96\sqrt{\left(\frac{1}{a}+\frac{1}{b}+\frac{1}{c}+\frac{1}{d}\right)} \end{matrix}$ | a≥3 with a lower 95% CI > 1 |
| PRR | $\begin{matrix} PRR=\frac{\text{a}/\left(a+b\right)}{c/(c+d)} \\ SE(InPRR)=\sqrt{\frac{1}{a}-\frac{1}{a+b}+\frac{1}{c}-\frac{1}{c+d}} \\ 95\%CI=eIn(PRR)\pm1.96\sqrt{(\frac{1}{a}-\frac{1}{a+b}+\frac{1}{c}-\frac{1}{c+d})} \end{matrix}$ | a≥3 with a lower 95% CI > 1 |

Abbreviation: a number of reports containing both the target drug and target adverse drug reaction; b, number of reports containing other adverse drug reaction of the target drug; c, number of reports containing the target adverse drug reaction of other drugs; d, number of reports containing other drugs and other adverse drug reactions. 95%CI, 95% confidence interval; N, the number of reports.

**Supplementary Table 3** Nervous system disorders adverse events for BTK inhibitors at the PT level in acalabrutinib from FAERS data.

| PT | Case number | ROR (95%Cl) | PRR (XX) |
| --- | --- | --- | --- |
| - Dementia | 25 | 3.29(2.22-4.88) | 3.29(39.83) |
| - Headache | 446 | 2.55(2.32-2.8) | 2.51(408.04) |
| - Brain fog | 19 | 8.49(5.41-13.32) | 8.48(125.06) |
| - Sleep deficit | 3 | 6.26(2.02-19.43) | 6.26(13.23) |
| - Cluster headache | 3 | 7.87(2.53-24.43) | 7.87(17.93) |
| - Polyneuropathy | 7 | 2.12(1.01-4.46) | 2.12(4.16) |
| - Carotid artery occlusion | 4 | 3.75(1.41-10) | 3.75(8.05) |
| - Brain stem haemorrhage | 3 | 10.84(3.49-33.69) | 10.84(26.71) |
| - Cerebral haemorrhage | 21 | 2.04(1.33-3.13) | 2.04(11.14) |
| - Transient ischaemic attack | 21 | 2.12(1.38-3.25) | 2.12(12.36) |
| - Dementia Alzheimer's type | 7 | 2.62(1.25-5.51) | 2.62(7.02) |

ROR, reporting odds ratio; PRR, proportional reporting ratio; CI, confidence interval; AEs, adverse events; PT, preferred term.

**Supplementary Table 4** Nervous system disorders adverse events for BTK inhibitors at the PT level in ibrutinib from FAERS data.

| PT | Case number | ROR (95%Cl) | PRR (XX) |
| --- | --- | --- | --- |
| - Central nervous system haemorrhage | 25 | 16.64(11.11-24.92) | 16.64(345.92) |
| - Spinal cord haemorrhage | 9 | 6.84(3.53-13.26) | 6.84(43.76) |
| - Post herpetic neuralgia | 26 | 5.41(3.67-7.98) | 5.41(91.58) |
| - Meningorrhagia | 4 | 4.44(1.65-11.93) | 4.44(10.49) |
| - Motor neurone disease | 7 | 4.4(2.08-9.28) | 4.4(18.07) |
| - Subdural hygroma | 5 | 4.19(1.73-10.14) | 4.19(11.97) |
| - Thrombotic stroke | 9 | 3.5(1.82-6.76) | 3.5(15.9) |
| - Cerebral haemorrhage | 419 | 3.49(3.17-3.85) | 3.49(734.19) |
| - Transient global amnesia | 8 | 3.27(1.63-6.57) | 3.27(12.47) |
| - Haemorrhage intracranial | 166 | 3.18(2.73-3.71) | 3.18(245.1) |
| - Intracranial mass | 13 | 3.03(1.75-5.23) | 3.03(17.48) |
| - Cerebral haematoma | 31 | 2.71(1.9-3.86) | 2.71(33.19) |
| - Bell's palsy | 11 | 2.58(1.43-4.68) | 2.58(10.58) |
| - Subarachnoid haemorrhage | 77 | 2.21(1.77-2.77) | 2.21(50.8) |
| - Carotid artery disease | 10 | 2.2(1.18-4.1) | 2.2(6.49) |
| - Metabolic encephalopathy | 22 | 2.18(1.43-3.32) | 2.18(13.98) |
| - Nerve compression | 62 | 2.04(1.59-2.62) | 2.04(32.58) |
| - Haemorrhagic stroke | 55 | 2.04(1.56-2.65) | 2.03(28.73) |

ROR, reporting odds ratio; PRR, proportional reporting ratio; CI, confidence interval; AEs, adverse events; PT, preferred term.

**Supplementary Table 5.** Nervous system disorders adverse events for BTK inhibitors at the PT level in zanubrutinib from FAERS data.

| PT | Case number | ROR (95%Cl) | PRR (XX) |
| --- | --- | --- | --- |
| Haemorrhagic stroke | 3 | 4.43(1.43-13.74) | 4.43(7.96) |
| Haemorrhage intracranial | 4 | 3.04(1.14-8.11) | 3.04(5.48) |
| Cerebral haemorrhage | 7 | 2.31(1.1-4.85) | 2.31(5.21) |

ROR, reporting odds ratio; PRR, proportional reporting ratio; CI, confidence interval; AEs, adverse events; PT, preferred term.

**Supplementary Table 6**. Comparison between the serious and non-serious groups for BTK inhibitor-related nervous system disorders AEs.

| PT | Serious case | NO-Serious case | pvalue | XX |
| --- | --- | --- | --- | --- |
| Encephalopathy | 33(0.028) | 31(0.0024) | <0.05 | 155.37 |
| Haemorrhage intracranial | 58(0.049) | 118(0.0092) | <0.05 | 139.34 |
| Cerebral haemorrhage | 98(0.084) | 349(0.027) | <0.05 | 109.41 |
| Unresponsive to stimuli | 21(0.018) | 25(0.002) | <0.05 | 83.06 |
| Ischaemic stroke | 26(0.022) | 45(0.0035) | <0.05 | 73.69 |
| Haemorrhagic stroke | 22(0.019) | 39(0.0031) | <0.05 | 60.80 |
| Dizziness | 61(0.052) | 1629(0.13) | <0.05 | 57.59 |
| Cerebrovascular accident | 114(0.097) | 599(0.047) | <0.05 | 55.97 |
| Central nervous system haemorrhage | 13(0.011) | 15(0.0012) | <0.05 | 52.62 |
| Metabolic encephalopathy | 11(0.0094) | 11(0.00086) | <0.05 | 49.46 |
| Cerebral infarction | 13(0.011) | 20(0.0016) | <0.05 | 41.21 |
| Headache | 81(0.069) | 1673(0.13) | <0.05 | 37.50 |
| Toxic encephalopathy | 6(0.0051) | 4(0.00031) | <0.05 | 34.56 |
| Balance disorder | 6(0.0051) | 391(0.031) | <0.05 | 25.26 |
| Memory impairment | 16(0.014) | 546(0.043) | <0.05 | 23.55 |
| Neurological symptom | 7(0.006) | 9(0.00071) | <0.05 | 25.96 |
| Hepatic encephalopathy | 4(0.0034) | 2(0.00016) | <0.05 | 26.44 |
| Vasogenic cerebral oedema | 3(0.0026) | 1(7.8e-05) | <0.05 | 23.02 |
| Neuropathy peripheral | 19(0.016) | 480(0.038) | <0.05 | 14.25 |
| Subarachnoid haemorrhage | 16(0.014) | 65(0.0051) | <0.05 | 13.60 |
| Cerebral haematoma | 9(0.0077) | 26(0.002) | <0.05 | 13.63 |
| Intraventricular haemorrhage | 4(0.0034) | 5(0.00039) | <0.05 | 15.17 |
| Brain injury | 8(0.0068) | 22(0.0017) | <0.05 | 13.00 |
| Altered state of consciousness | 5(0.0043) | 9(0.00071) | <0.05 | 13.56 |
| Hypoaesthesia | 16(0.014) | 383(0.03) | <0.05 | 10.33 |
| Amnesia | 5(0.0043) | 209(0.016) | <0.05 | 10.41 |
| Hypoxic-ischaemic encephalopathy | 3(0.0026) | 3(0.00024) | <0.05 | 13.47 |
| Leukoencephalopathy | 3(0.0026) | 3(0.00024) | <0.05 | 13.47 |
| Depressed level of consciousness | 7(0.006) | 20(0.0016) | <0.05 | 10.77 |
| Paraesthesia | 9(0.0077) | 251(0.02) | <0.05 | 8.43 |
| Coma | 14(0.012) | 66(0.0052) | <0.05 | 8.62 |
| Brain stem infarction | 2(0.0017) | 1(7.8e-05) | <0.05 | 13.22 |
| Migraine | 2(0.0017) | 117(0.0092) | <0.05 | 7.06 |
| Loss of consciousness | 33(0.028) | 557(0.044) | <0.05 | 6.36 |
| Cerebral ischaemia | 4(0.0034) | 9(0.00071) | <0.05 | 8.44 |
| Dementia | 24(0.02) | 155(0.012) | <0.05 | 5.87 |
| Burning sensation | 3(0.0026) | 118(0.0092) | <0.05 | 5.58 |
| Cognitive disorder | 15(0.013) | 86(0.0067) | <0.05 | 5.47 |
| Spinal cord haemorrhage | 3(0.0026) | 6(0.00047) | <0.05 | 7.26 |
| Carpal tunnel syndrome | 1(0.00085) | 75(0.0059) | <0.05 | 5.00 |
| Tremor | 16(0.014) | 297(0.023) | <0.05 | 4.53 |
| Lethargy | 18(0.015) | 118(0.0092) | 0.06 | 4.15 |
| Ataxia | 4(0.0034) | 13(0.001) | 0.07 | 5.05 |
| Nerve compression | 1(0.00085) | 65(0.0051) | 0.07 | 4.09 |
| Aphasia | 14(0.012) | 87(0.0068) | 0.07 | 3.92 |
| Brain stem haemorrhage | 2(0.0017) | 3(0.00024) | 0.08 | 6.48 |
| Cerebrovascular disorder | 2(0.0017) | 3(0.00024) | 0.08 | 6.48 |
| Haemorrhagic transformation stroke | 2(0.0017) | 3(0.00024) | 0.08 | 6.48 |
| Ischaemic cerebral infarction | 2(0.0017) | 3(0.00024) | 0.08 | 6.48 |
| Neurological decompensation | 2(0.0017) | 3(0.00024) | 0.08 | 6.48 |
| Central nervous system lesion | 7(0.006) | 34(0.0027) | 0.09 | 4.00 |
| Somnolence | 21(0.018) | 340(0.027) | 0.09 | 3.24 |
| Speech disorder | 4(0.0034) | 109(0.0085) | 0.09 | 3.51 |
| Hypersomnia | 5(0.0043) | 121(0.0095) | 0.10 | 3.26 |
| Hydrocephalus | 4(0.0034) | 15(0.0012) | 0.12 | 3.94 |
| Sciatica | 4(0.0034) | 102(0.008) | 0.12 | 2.98 |
| Bell's palsy | 3(0.0026) | 9(0.00071) | 0.12 | 4.29 |
| Dyskinesia | 5(0.0043) | 22(0.0017) | 0.12 | 3.59 |
| Cerebellar haemorrhage | 2(0.0017) | 4(0.00031) | 0.14 | 4.84 |
| Myasthenia gravis | 2(0.0017) | 4(0.00031) | 0.14 | 4.84 |
| Hemiparesis | 6(0.0051) | 31(0.0024) | 0.16 | 2.93 |
| Ageusia | 2(0.0017) | 66(0.0052) | 0.16 | 2.66 |
| Dizziness postural | 1(0.00085) | 50(0.0039) | 0.16 | 2.77 |
| Brain fog | 1(0.00085) | 49(0.0038) | 0.17 | 2.68 |
| Motor dysfunction | 3(0.0026) | 11(0.00086) | 0.20 | 3.08 |
| Peripheral sensory neuropathy | 3(0.0026) | 11(0.00086) | 0.20 | 3.08 |
| Carotid artery stenosis | 3(0.0026) | 12(0.00094) | 0.25 | 2.62 |
| Hypokinesia | 3(0.0026) | 12(0.00094) | 0.25 | 2.62 |
| Neurotoxicity | 3(0.0026) | 12(0.00094) | 0.25 | 2.62 |
| Dysgeusia | 7(0.006) | 122(0.0096) | 0.29 | 1.51 |
| Epilepsy | 1(0.00085) | 37(0.0029) | 0.32 | 1.65 |
| Syncope | 27(0.023) | 360(0.028) | 0.35 | 1.07 |
| Hemiplegia | 3(0.0026) | 14(0.0011) | 0.35 | 1.88 |
| Paraplegia | 2(0.0017) | 7(0.00055) | 0.37 | 2.23 |
| Thrombotic stroke | 2(0.0017) | 7(0.00055) | 0.37 | 2.23 |
| Dysstasia | 8(0.0068) | 58(0.0045) | 0.39 | 1.18 |
| Arachnoiditis | 1(0.00085) | 1(7.8e-05) | 0.40 | 4.49 |
| Cerebellar infarction | 1(0.00085) | 1(7.8e-05) | 0.40 | 4.49 |
| Diabetic hyperosmolar coma | 1(0.00085) | 1(7.8e-05) | 0.40 | 4.49 |
| Essential tremor | 1(0.00085) | 1(7.8e-05) | 0.40 | 4.49 |
| Meningeal disorder | 1(0.00085) | 1(7.8e-05) | 0.40 | 4.49 |
| Paralysis | 2(0.0017) | 42(0.0033) | 0.51 | 0.86 |
| Movement disorder | 4(0.0034) | 26(0.002) | 0.52 | 0.94 |
| Presyncope | 3(0.0026) | 54(0.0042) | 0.54 | 0.74 |
| Transient ischaemic attack | 18(0.015) | 233(0.018) | 0.55 | 0.51 |
| Parkinson's disease | 4(0.0034) | 66(0.0052) | 0.55 | 0.66 |
| Facial paralysis | 7(0.006) | 55(0.0043) | 0.56 | 0.67 |
| Anosmia | 1(0.00085) | 26(0.002) | 0.59 | 0.78 |
| Cerebral disorder | 4(0.0034) | 28(0.0022) | 0.61 | 0.70 |
| Brain hypoxia | 1(0.00085) | 2(0.00016) | 0.61 | 2.42 |
| Consciousness fluctuating | 1(0.00085) | 2(0.00016) | 0.61 | 2.42 |
| Diplegia | 1(0.00085) | 2(0.00016) | 0.61 | 2.42 |
| Encephalomalacia | 1(0.00085) | 2(0.00016) | 0.61 | 2.42 |
| Intention tremor | 1(0.00085) | 2(0.00016) | 0.61 | 2.42 |
| Intracranial tumour haemorrhage | 1(0.00085) | 2(0.00016) | 0.61 | 2.42 |
| Neuromyopathy | 1(0.00085) | 2(0.00016) | 0.61 | 2.42 |
| Post herpetic neuralgia | 1(0.00085) | 25(0.002) | 0.63 | 0.71 |
| Mental impairment | 5(0.0043) | 38(0.003) | 0.63 | 0.58 |
| Nervous system disorder | 8(0.0068) | 68(0.0053) | 0.65 | 0.44 |
| Brain oedema | 4(0.0034) | 29(0.0023) | 0.65 | 0.59 |
| Multiple sclerosis | 1(0.00085) | 24(0.0019) | 0.66 | 0.63 |
| Sensory disturbance | 1(0.00085) | 24(0.0019) | 0.66 | 0.63 |
| Dementia alzheimer's type | 5(0.0043) | 39(0.0031) | 0.66 | 0.50 |
| Spinal cord compression | 2(0.0017) | 11(0.00086) | 0.68 | 0.82 |
| Intracranial mass | 2(0.0017) | 12(0.00094) | 0.76 | 0.63 |
| Embolic cerebral infarction | 1(0.00085) | 3(0.00024) | 0.77 | 1.43 |
| Meningorrhagia | 1(0.00085) | 3(0.00024) | 0.77 | 1.43 |
| Quadriparesis | 1(0.00085) | 3(0.00024) | 0.77 | 1.43 |
| Senile dementia | 1(0.00085) | 3(0.00024) | 0.77 | 1.43 |
| Incoherent | 3(0.0026) | 23(0.0018) | 0.83 | 0.33 |
| Disturbance in attention | 4(0.0034) | 53(0.0042) | 0.89 | 0.14 |
| Cervical radiculopathy | 1(0.00085) | 4(0.00031) | 0.90 | 0.87 |
| Iiird nerve paralysis | 1(0.00085) | 4(0.00031) | 0.90 | 0.87 |
| Lacunar infarction | 1(0.00085) | 4(0.00031) | 0.90 | 0.87 |
| Lumbar radiculopathy | 1(0.00085) | 4(0.00031) | 0.90 | 0.87 |
| Status epilepticus | 1(0.00085) | 4(0.00031) | 0.90 | 0.87 |
| Subdural hygroma | 1(0.00085) | 4(0.00031) | 0.90 | 0.87 |
| Dysarthria | 6(0.0051) | 59(0.0046) | 0.99 | 0.06 |
| Hyperaesthesia | 1(0.00085) | 17(0.0013) | 0.99 | 0.19 |
| Posterior reversible encephalopathy syndrome | 1(0.00085) | 17(0.0013) | 0.99 | 0.19 |
| Seizure | 30(0.026) | 321(0.025) | 1.00 | 0.01 |
| Polyneuropathy | 3(0.0026) | 36(0.0028) | 1.00 | 0.03 |
| Monoplegia | 1(0.00085) | 12(0.00094) | 1.00 | 0.01 |
| Cerebral thrombosis | 1(0.00085) | 11(0.00086) | 1.00 | 0.00 |
| Generalised tonic-clonic seizure | 1(0.00085) | 11(0.00086) | 1.00 | 0.00 |
| Parosmia | 3(0.0026) | 27(0.0021) | 1.00 | 0.10 |
| Amyotrophic lateral sclerosis | 1(0.00085) | 8(0.00063) | 1.00 | 0.09 |
| Chronic inflammatory demyelinating polyradiculoneuropathy | 1(0.00085) | 7(0.00055) | 1.00 | 0.17 |
| Coordination abnormal | 1(0.00085) | 9(0.00071) | 1.00 | 0.03 |
| Demyelinating polyneuropathy | 1(0.00085) | 6(0.00047) | 1.00 | 0.31 |
| Drooling | 1(0.00085) | 5(0.00039) | 1.00 | 0.53 |
| Embolic stroke | 1(0.00085) | 7(0.00055) | 1.00 | 0.17 |
| Fine motor skill dysfunction | 1(0.00085) | 5(0.00039) | 1.00 | 0.53 |
| Paraparesis | 1(0.00085) | 7(0.00055) | 1.00 | 0.17 |
| Parkinsonism | 1(0.00085) | 6(0.00047) | 1.00 | 0.31 |
| Radiculopathy | 1(0.00085) | 7(0.00055) | 1.00 | 0.17 |
| Tongue biting | 1(0.00085) | 8(0.00063) | 1.00 | 0.09 |

PT, preferred term.
